# What We Know About Tuberculosis Transmission: An Overview

**DOI:** 10.1093/infdis/jix362

**Published:** 2017-11-03

**Authors:** Gavin Churchyard, Peter Kim, N Sarita Shah, Roxana Rustomjee, Neel Gandhi, Barun Mathema, David Dowdy, Anne Kasmar, Vicky Cardenas

**Affiliations:** 1 Aurum Institute, Johannesburg, South Africa,; 2 School of Public Health, University of the Witwatersrand, Johannesburg, South Africa,; 3 Advancing Care & Treatment for TB/HIV, Johannesburg, South Africa, and; 4 South African Medical Research Council, Johannesburg, South Africa;; 5 Division of AIDS, National Institutes of Health, Bethesda, Maryland, and; 6 Johns Hopkins University, Baltimore, Maryland;; 7 Division of Global HIV and Tuberculosis, Centers for Disease Control and Prevention, Atlanta, Georgia, and; 8 Rollins School of Public Health, Emory University, Atlanta, Georgia, and; 9 Emory School of Medicine, Emory University, Atlanta, Georgia;; 10 Department of Epidemiology, Mailman School of Public Health, Columbia University, New York, New York; and; 11 Bill and Melinda Gates Foundation, Seattle, Washington

**Keywords:** Tuberculosis, transmission

## Abstract

Tuberculosis remains a global health problem with an enormous burden of disease, estimated at 10.4 million new cases in 2015. To stop the tuberculosis epidemic, it is critical that we interrupt tuberculosis transmission. Further, the interventions required to interrupt tuberculosis transmission must be targeted to high-risk groups and settings. A simple cascade for tuberculosis transmission has been proposed in which (1) a source case of tuberculosis (2) generates infectious particles (3) that survive in the air and (4) are inhaled by a susceptible individual (5) who may become infected and (6) then has the potential to develop tuberculosis. Interventions that target these events will interrupt tuberculosis transmission and accelerate the decline in tuberculosis incidence and mortality. The purpose of this article is to provide a high-level overview of what is known about tuberculosis transmission, using the tuberculosis transmission cascade as a framework, and to set the scene for the articles in this series, which address specific aspects of tuberculosis transmission.

Tuberculosis remains a global health problem with an enormous burden of disease, estimated at 10.4 million new cases in 2015, of which 10% were among children and 12% involved human immunodeficiency virus (HIV) coinfection [1]. In 2015, there were an estimated 1.8 million deaths due to tuberculosis, including HIV-associated tuberculosis deaths, making tuberculosis the leading cause of death from an infectious disease [1]. Latent *Mycobacterium tuberculosis* infection is the reservoir of the tuberculosis epidemic. The global burden of *M. tuberculosis* infection has recently been reestimated at 24% [2].

The global rate of decline in tuberculosis incidence is currently 1.5% and will need to increase to 4%–5% by 2020 and then to 10% per year by 2025 to meet the World Health Organization End TB Strategy targets (Figure 1)[3]. Interrupting tuberculosis transmission is central to achieving the reductions in tuberculosis incidence required to meet the End TB targets. A rate of decline of 10% per year is thought to be achievable, as this was observed during the 1950s and 1960s in Western Europe, where comprehensive tuberculosis control efforts, which included infection control and treatment of *M. tuberculosis* infection and all forms of tuberculosis, were intensified and universal health coverage and socioeconomic development were expanded [1]. Specific examples include declines in tuberculosis mortality and incidence observed after the second world war in England, Wales, and the Netherlands, where improved socioeconomic conditions and better nutrition and living standards were thought to be major factors contributing to improved tuberculosis control [4]. Tuberculosis case notifications among children <5 years of age declined in New York and London but not in Cape Town over the century between 1912 and 2012 despite similar, contemporaneous tuberculosis control strategies, which included the introduction of chemotherapy in the mid-1950s. This observation suggests that socioeconomic development played a greater role than the introduction of chemotherapy in reducing tuberculosis transmission [5]. Mathematical modeling suggests that it is possible to rapidly reduce tuberculosis incidence and mortality in high-burden countries, including those with a high HIV prevalence, if a comprehensive strategy of combination treatment and prevention is implemented at scale, rapidly [6]. Modeling further suggests that development of new drugs, diagnostic assays, and vaccines will be essential to accelerate progress toward tuberculosis elimination [7]. We have a unique opportunity in the era of the United Nations Sustainable Development Goals to address poverty and other social determinants of tuberculosis while simultaneously scaling up currently available effective tuberculosis control interventions to interrupt tuberculosis transmission and thereby maximize impact on reducing tuberculosis incidence and mortality. In addition, we should continue to conduct research to optimize delivery of effective interventions, as well as develop new tools that can maximize interrupting tuberculosis transmission.

**Figure 1.**
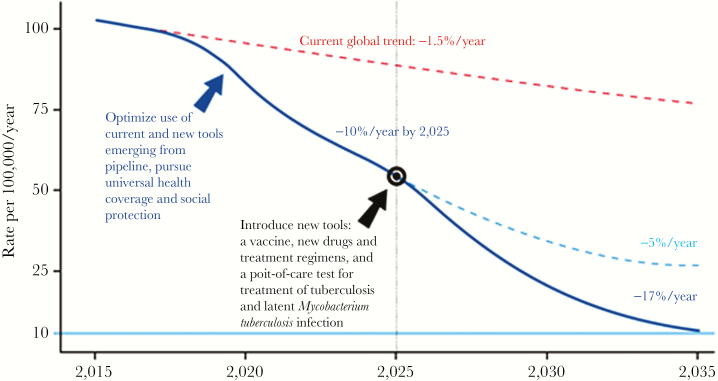
Projected acceleration in the decline of global tuberculosis incidence rates to target levels. From WHO END TB Strategy [3].

In March 2016, the National Institutes of Health convened a workshop aimed at identifying the research needs for halting tuberculosis transmission, with the eventual aim of reducing new *M. tuberculosis* infections to zero. The purpose of this article is to give a high-level overview of the discussion at the workshop regarding what is known about tuberculosis transmission and to set the scene for the articles that address specific aspects of tuberculosis transmission. The lessons learned from studying tuberculosis transmission are also relevant to reducing transmission of other airborne pathogens.

## EPIDEMIOLOGY OF TUBERCULOSIS TRANSMISSION: A BRIEF HISTORY

Robert Koch discovered *M. tuberculosis* in 1882. William Osler, in 1909, wrote that “all who mix with tuberculosis patients got infected, but remained well so long as they took care of themselves and kept the soil in a condition unfavorable for the growth of the seed” [8]. Over the intervening century of tuberculosis research, our understanding of tuberculosis transmission and disease progression has improved: in 1920, Devoto recognized that healthcare workers were at risk of developing tuberculosis; in 1934, Wells described the falling and evaporation times for droplet nuclei [9]; and Riley, in 1961, described the deposition of airborne bacteria in the lung [10] and, in 1960–1962, described aerial dissemination of *M. tuberculosis* in a tuberculosis ward [11, 12]. Chapman, in 1964, described the social and other factors associated with tuberculosis transmission in tuberculosis-affected households [13].

More recent achievements (circa mid-2000s) in this area include the phylogeographical classification of global *M. tuberculosis* strains and the advent of whole-genome sequencing for molecular tracking of tuberculosis outbreaks.

## TUBERCULOSIS TRANSMISSION CASCADE

**Figure 2.**
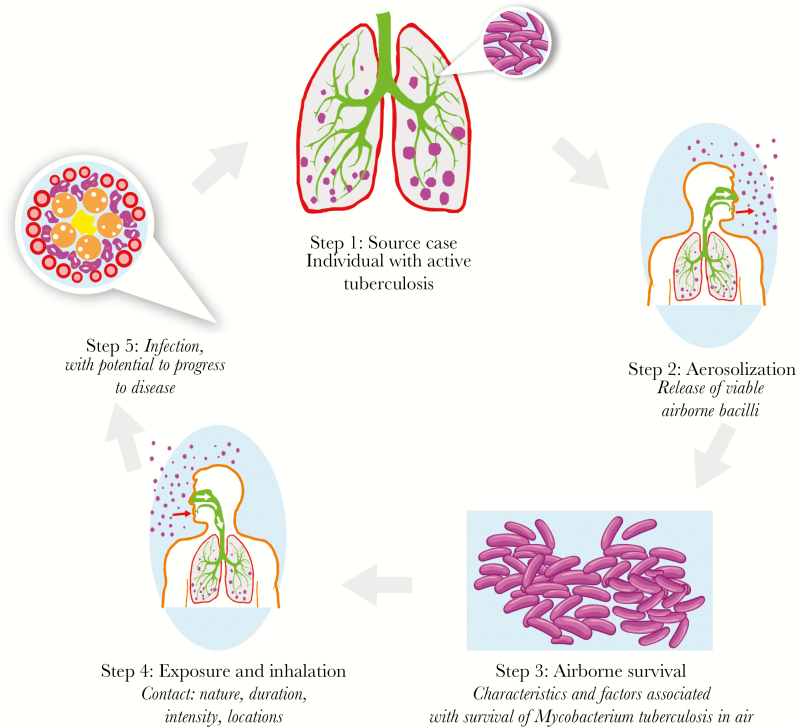
Cascade of tuberculosis transmission. (Source: The Aurum Institute)

In this series, a simple cascade for tuberculosis transmission is proposed in which (1) a source case of tuberculosis (2) generates infectious particles (3) that survive in the air and (4) are inhaled by a susceptible individual (5) who may become infected and (6) who then has the potential to develop tuberculosis. Interventions that target bacterial, host, or behavioral catalysts of transmission will interrupt tuberculosis transmission and accelerate the decline in tuberculosis incidence and mortality [14]. In this article, this cascade of tuberculosis transmission will be used to describe who is transmitting, where transmission is occurring, and who is susceptible to infection and to disease progression. In answering these questions, we can understand what it will take to stop tuberculosis transmission (Figure 2).

## WHO IS TRANSMITTING?

The infectiousness and duration thereof for a person with tuberculosis depend on host and bacterial factors. Persons with smear-positive pulmonary tuberculosis are highly infectious, and the degree of infectiousness is thought to increase with the degree of smear positivity. In a large study of household contacts in Peru, smear-positive index cases were associated with a higher risk of infection among household contacts, compared with smear-negative index cases, regardless of the age of the household contacts [15]. Persons with smear-negative tuberculosis cases may, however, also transmit tuberculosis [16]. Nevertheless, scale-up of sputum smear microscopy has not succeeded in achieving dramatic declines in tuberculosis incidence. Possible reasons for the lack of impact include the poor sensitivity of smear microscopy, particularly among HIV-infected persons and children, and the occurrence of many cases of transmission before people receive a tuberculosis diagnosis and treatment.

Persons with active pulmonary or laryngeal tuberculosis generate droplet nuclei that contain *M. tuberculosis* through coughing, singing, shouting, sneezing, or any other forceful expiratory maneuver that shears respiratory secretions from the airways, with coughing being the most efficient at generating infectious aerosols [16]. Appropriate treatment of individuals with infectious tuberculosis results in a rapid reduction in infectiousness [17]. Individuals with index tuberculosis cases who are HIV infected, particularly those with advanced immunosuppression, were hypothesized to be less likely than HIV-uninfected individuals with tuberculosis to transmit to household contacts, possibly because of a greater likelihood of having smear-negative tuberculosis and a shorter duration of infectiousness due to more rapid progression to death [18, 19].

Antiretroviral therapy reduces the risk of tuberculosis among people with HIV infection (PLHIV) by 67% and, if scaled up, may contribute to a reduction in tuberculosis case rates at a population level [20, 21]. Although there is preferential mixing of close contacts within age groups and sexes, in Southern Africa most *M. tuberculosis* infections appear to be associated with contact with adult men [22].

## WHERE IS TRANSMISSION OCCURRING?

Robert Koch, in his Nobel Lecture, delivered in 1905, said that “tuberculosis has been called plainly, and quite justly, a disease of accommodation” [23], highlighting transmission of tuberculosis within tuberculosis-affected households. Today there is a wealth of evidence to support transmission of drug-susceptible and drug-resistant tuberculosis in households [15, 24, 25]. Transmission of tuberculosis to household contacts is most likely to occur when the index case is smear positive and the household contacts are <15 years of age [15, 26]. Despite a historical focus on household transmission, the overall proportion of tuberculosis transmissions that occur in households is estimated to be between 8% and 19% in countries with a high HIV prevalence, such as South Africa and Malawi [27]. In settings with a high tuberculosis burden, tuberculosis transmission is therefore more likely to occur outside the household, in schools, public transportation settings, workplaces, healthcare facilities, mines, and prisons [19, 26–33]. Nevertheless, targeting tuberculosis-affected households for tuberculosis screening, HIV testing, and referral for treatment of tuberculosis or *M. tuberculosis* infection remains a priority because of the high prevalence of tuberculosis and *M. tuberculosis* infection among household contacts. Transmission within hospitals and clinics can be reduced by using the FAST approach: *F*inding undiagnosed tuberculosis cases *A*ctively through cough surveillance and use of rapid molecular diagnostics, *S*eparating safely, and providing appropriate *T*reatment [34]. Geographic areas with increased tuberculosis transmission (so-called hot spots) may be identified using geospatial mapping, and interventions targeted to these areas may help to interrupt transmission [25]. In a country with a low tuberculosis burden, such as the United States, targeting active, community-based screening and isoniazid preventive therapy to 2 relatively high-burden neighborhoods was effective in eliminating tuberculosis in the intervention neighborhoods [35].

## WHO IS SUSCEPTIBLE?

Close contacts of infectious tuberculosis cases are susceptible to becoming infected and, if infected, to progressing to tuberculosis, particularly within the first year after exposure [36]. Among 95 contact investigation studies from countries of low and middle incomes, the prevalence of *M. tuberculosis* infection among contacts was 51.5%. Contacts who are <5 years of age or HIV infected have the greatest risk of developing tuberculosis [36]. Among countries with high burdens of tuberculosis and HIV infection, such as South Africa and Zambia, HIV-infected household contacts have a risk of progressing to tuberculosis that is almost 5-fold greater than that for HIV-uninfected household contacts [37]. In settings with a high tuberculosis burden, silica-exposed miners, particularly those with silicosis, have a high prevalence of *M. tuberculosis* infection [38, 39]. Many persons who are at high risk of developing tuberculosis can be identified on the basis of their medical history or with simple tests [14]. Currently, it is not possible to identify persons who have an increased risk of infection if exposed.

## HALTING TRANSMISSION

Halting tuberculosis transmission is central to stopping the tuberculosis epidemic. As shown in Figure 2, it may be possible to target interventions to reduce the infectiousness or duration of infectiousness of tuberculosis cases, contact rates, and susceptibility of contacts. Contact between an infectious tuberculosis case and a susceptible person may occur because of clustering in space (such as in households, workplaces, and urban slums) or over time (such as in public transportation settings, among migrant workers, and during urbanization or displacement) [14]. Contact rates can be reduced through socioeconomic development leading to reduced crowding. Socioeconomic development also improves nutrition, reducing progression to tuberculosis. Improved infection control also accompanies socioeconomic development, particularly improved ventilation in areas where contact is likely to occur, such as healthcare facilities, public transportation settings, workplaces, and schools [5, 14]. Infectiousness and the duration of infectiousness can be reduced through early case detection and treatment by improving access to quality tuberculosis diagnostic and treatment services, use of quicker and more-sensitive diagnostic assays such as Xpert MTB/Rif, active case finding and linkage to care for appropriate treatment, and interventions to reduce attrition before starting treatment [6]. Susceptibility to tuberculosis can be reduced by addressing host factors such as HIV infection, diabetes, anti–tumor necrosis factor treatment, organ transplantation, renal dialysis, silicosis, illicit drug use, malnutrition, harmful alcohol use, and smoking. Susceptibility to tuberculosis can also be reduced by treating the underlying condition (eg, HIV infection and diabetes), by reducing key exposures (eg, silica dust, tobacco smoke, and indoor pollutants), and by providing preventive therapy for latent *M. tuberculosis* infection.

A number of intervention studies have attempted to reduce tuberculosis transmission at a population level by using combinations of case finding and preventive therapy interventions targeting at-risk groups or communities, with success ranging from no or minimal impact to large and sustained impact (Table 1) [40–51]. The variable success of the interventions in achieving a population-level impact may be due to poor targeting of risk groups, inadequate coverage, implementation of interventions that are not implemented or evaluated long enough to capture mass effect, and use of old tools, such as sputum microscopy.

**Table 1.** Studies Evaluating Tuberculosis Interventions Intended to Achieve a Population-Level Impact

Country(ies), Year, Reference(s)	Setting	Design(s)	Intervention(s)	Outcome Measure(s)	Finding(s)
Tunisia, 1963 [41, 42]	Urban slums (n = 153)	CRT (housing blocks)	IPT for 12 mo	Tuberculosis case rates (cases/1000)	2.3 cases/1000 in IPT arm vs 3.1 in placebo arm (25.8% reduction)
Greenland, 1966 [42–44]	Villages (n = 76)	CRT (villages)	2 courses of INH 400 mg twice weekly for 3 mo, 3 mo apart	Cumulative case rates	5.7% in IPT arm vs 8.3% in placebo arm (31.3% reduction)
US, 1967 [40, 41, 44]	Alaska, Bethel communities (n = 30)	CRT (households)	Household-wide IPT for 12 mo	Cumulative case rate	1.90% in IPT arm vs 4.67% in placebo arm (59.3% reduction)
US, 1986 [46]	Oregon, Burnside area	Before/after intervention (homeless shelters)	Mandatory tuberculosis screening and treatment of tuberculosis or *M. tuberculosis* infection among persons using homeless shelter	Case notification rate, 1995 vs 1985	29 cases/100 000 in 1995 vs 227 in 1985 (decline greater than that observed in other districts)
Zimbabwe, 2005 [47]	Harare, high-density suburbs (n = 46)	CRT (suburbs)	Tuberculosis screening via mobile van or door to door	Tuberculosis prevalence; before vs after intervention for both arms combined	6.5 cases/1000 at baseline vs 3.7 after intervention (aRR, 0.59 [95% CI, .40–.89])
Zambia and South Africa, 2006 [48]	Communities in South Africa and Zambia (n = 24)	CRT (communities), factorial design	(1) ECF vs non-ECF; (2) household care vs non– household care	(1) Tuberculosis prevalence, infection incidence; (2) tuberculosis prevalence, infection incidence	(1) 927 cases of tuberculosis/100 000 in ECF arm vs 711 in non-ECF arm (aRR, 1.11 [95% CI, .87–1.42]); 1.41% infection incidence in ECF arm vs 1.05% in non-ECF arm (aRR, 1.36 [95% CI, .59–3.14]); (2) 746 cases of tuberculosis/100 000 in household care arm vs 833 in non–household care arm (aRR, 0.78 [95% CI, .61–1.00]); 0.87% infection incidence in household care arm vs 1.71% in non–household care arm (RR, 1.36 [95% CI, .59–3.14])
Brazil, 2010 [49]	Urban communities (n = 8)	CRT (favelas)	Tuberculosis screening plus IPT in household contacts	Tuberculosis incidence	358 cases/100 000 in control arm vs 305 in intervention arm (*P* = 0.04)
South Africa, 2011 [29]	Gold mines (n = 16 clusters)	CRT (mines)	Community-wide tuberculosis screening and IPT	Tuberculosis incidence	3.02 cases/100 person-years in intervention arm vs 2.95 in control arm (aRR, 0.96 [95% CI, .76–1.21])
Brazil, 2013 [50]	Rio de Janeiro, HIV clinics (n = 29)	CRT (HIV clinics), step wedge	IPT promotion	Incidence of tuberculosis alone, incidence of tuberculosis and death	1.1 cases of tuberculosis/100 person-years in intervention arm vs 1.31 in control arm (aHR, 0.73 [95% CI, .54–.99]); 3.04 cases of tuberculosis and deaths/100 person-years vs 3.64 in control arm (aHR, 0.69 [95% CI, .57–.83])

Data are adapted and expanded from the article by Kranzer et al [51], which used a nonsystematic literature review and was therefore not comprehensive.

Abbreviations: aHR, adjusted hazard ratio; aRR, adjusted rate ratio; CI, confidence interval; CRT, cluster randomized trial; ECF, enhanced case finding; HIV, human immunodeficiency virus; IPT, isoniazid preventive therapy; *M. tuberculosis*, *Mycobacterium tuberculosis*; RR, rate ratio.

## NEW TOOLS

Advances in technology may allow more-effective targeting of the sources of tuberculosis transmission. The Xpert MTB/RIF test was initially heralded as a “game changer” in the diagnosis of tuberculosis, owing to its greater sensitivity than sputum smear microscopy [52, 53]. The roll out of Xpert MTB/RIF, however, has had limited impact on tuberculosis mortality and incidence to date, largely because of health system weaknesses, particularly those due to poor uptake of HIV testing and linkage to care for antiretroviral therapy. This highlights the need to strengthen health systems and develop new tools [54, 55]. The next-generation Xpert MTB/Rif (Ultra) cartridge is expected to be even more sensitive and could be a valuable tool to identify active and infectious cases, thereby allowing the prevention of transmission. Modeling suggests that new tuberculosis drugs and regimens for drug-susceptible and drug-resistant tuberculosis that are shorter and more efficacious may have a modest population-level impact [7, 56]. New tests for individuals with *M. tuberculosis* infection that predict who will progress to tuberculosis will allow treatment of infection to be targeted to those at greatest risk of developing tuberculosis [57]. Implementation of new, short-course regimens for treating latent *M. tuberculosis* infection, such as weekly high-dose isoniazid and rifapentine for 3 months or daily isoniazid and rifampicin for 3 months, potentially could have a profound effect on the tuberculosis epidemic, particularly if implemented at scale and coupled with active case finding and treatment of all forms of tuberculosis. Barriers to scaling up treatment of *M. tuberculosis* infection should be addressed, and innovative, affordable models of delivery that support scale up of treatment of *M. tuberculosis* infection should be evaluated [58]. New research tools, such as whole-genome sequencing, could help us understand global and local tuberculosis epidemiology better and thereby target interventions to reduce transmission more effectively [59]. Similarly, tuberculosis vaccines that prevent *M. tuberculosis* infection or disease among adolescents and adults may have a profound impact on the tuberculosis epidemic [60].

## CONCLUSION

Robert Koch, in his Nobel Lecture, said that “amidst the persistently great variety in the ways and means of combating tuberculosis, it is yet necessary to ask what measures do indeed best satisfy the scientific requirements” [23]. More than 100 years later, we are still asking the same question. Although our understanding of tuberculosis transmission has improved substantially, many gaps remain. Subsequent articles in this series aim to identify these gaps and to describe the benefits (and obstacles) to filling them. One thing is certain: if we wish to end tuberculosis by 2035, a massive concerted effort is required today.

## Notes

***Financial support.*** This publication and workshop have been funded in whole or in part with Federal Funds from the Division of AIDS, National Institute of Allergy and Infectious Diseases (NIAID), US National Institutes of Health (NIH), Department of Health and Human Services under contract number HHSN272201100001G – Research Support Services for the Division of AIDS. Funding support was alsoprovided in part by NIH grants: NIAID R01AI089349 (PI Gandhi), NIAID R01AI087465 (PI Gandhi), NIAIDK24AI114444 (PI Gandhi), Emory CFAR P30AI050409 (PI JW Curran), and NHLBI T32 HL116271 (PI DM Guidot).The Bill & Melinda Gates Foundation provided funding for participant travel to the workshop. TheCenters for Disease Control and Prevention (CDC) and the South African Medical Research Council co-hostedthe event.

***Supplement sponsorship*** This work is part of a supplement sponsored by the Centers for Disease Control and Prevention and the National Institutes of Health.

***Potential conflicts of interest.*** All authors: No reported conflicts of interest. All authors have submitted the ICMJE Form for Disclosure of Potential Conflicts of Interest. Conflicts that the editors consider relevant to the content of the manuscript have been disclosed.

## References

[1] World Health Organization (WHO). Global tuberculosis report 2016. WHO/HTM/TB/2016.13. Geneva, Switzerland: WHO, 2016.

[2] Houben RM, Dodd PJ The global burden of latent tuberculosis infection: a re-estimation using mathematical modelling. PLoS Med 2016; 13:e1002152. 27780211 10.1371/journal.pmed.1002152 PMC5079585

[3] Barnagarwala T TB hospital staff live under shadow of dreaded disease. The Indian Express. Uttar Pradesh, India: IE Online Media Services, 2014.

[4] Lienhardt C, Glaziou P, Uplekar M, Lönnroth K, Getahun H, Raviglione M Global tuberculosis control: lessons learnt and future prospects. Nat Rev Microbiol 2012; 10:407–16. 22580364 10.1038/nrmicro2797

[5] Hermans S, Horsburgh CR Jr, Wood R A century of tuberculosis epidemiology in the Northern and Southern hemisphere: the differential impact of control interventions. PLoS One 2015; 10:e0135179. 26288079 10.1371/journal.pone.0135179 PMC4545605

[6] Houben RM, Menzies NA, Sumner T Feasibility of achieving the 2025 WHO global tuberculosis targets in South Africa, China, and India: a combined analysis of 11 mathematical models. Lancet Glob Health 2016; 4:e806–15. 27720688 10.1016/S2214-109X(16)30199-1 PMC6375908

[7] Abu-Raddad LJ, Sabatelli L, Achterberg JT Epidemiological benefits of more-effective tuberculosis vaccines, drugs, and diagnostics. Proc Natl Acad Sci U S A 2009; 106:13980–5. 19666590 10.1073/pnas.0901720106 PMC2720405

[8] Dobbs TE, Kimmerling ME. Mycobacterium tuberculosis. In AIDS Therapy E-Book. Philadelphia, PA: Elsevier, 2008.

[9] Wells WF On air-borne infection. Study II. Droplets and droplet nuclei. Am J Hyg. 1934:611–8.

[10] Riley RL, O’Grady F. Airborne infection: transmission and control. New York, NY: Macmillan, 1961.

[11] Riley RL, Mills CC, Nyka W Aerial dissemination of pulmonary tuberculosis: a two-year study of contagion in a tuberculosis ward. Am J Hyg 1959; 70:185–96. 10.1093/oxfordjournals.aje.a117542 7785671

[12] Riley RL, Mills CC, O’Grady F, Sultan LU, Wittstadt F, Shivpuri DN Infectiousness of air from a tuberculosis ward. Ultraviolet irradiation of infected air: comparative infectiousness of different patients. Am Rev Respir Dis 1962; 85:511–25. 14492300 10.1164/arrd.1962.85.4.511

[13] Chapman JS, Dyerly MD Social and other factors in intrafamilial transmission of tuberculosis. Am Rev Respir Dis 1964; 90:48–60. 14178626 10.1164/arrd.1964.90.1.48

[14] Dowdy DW, Azman AS, Kendall EA, Mathema B Transforming the fight against tuberculosis: targeting catalysts of transmission. Clin Infect Dis 2014; 59:1123–9. 24982034 10.1093/cid/ciu506 PMC4481585

[15] Zelner JL, Murray MB, Becerra MC Age-specific risks of tuberculosis infection from household and community exposures and opportunities for interventions in a high-burden setting. Am J Epidemiol 2014; 180:853–61. 25190676 10.1093/aje/kwu192 PMC4188339

[16] Turner RD, Bothamley GH Cough and the transmission of tuberculosis. J Infect Dis 2015; 211:1367–72. 25387581 10.1093/infdis/jiu625

[17] Dharmadhikari AS, Mphahlele M, Venter K Rapid impact of effective treatment on transmission of multidrug-resistant tuberculosis. Int J Tuberc Lung Dis 2014; 18:1019–25. 25189547 10.5588/ijtld.13.0834 PMC4692272

[18] Huang CC, Tchetgen ET, Becerra MC The effect of HIV-related immunosuppression on the risk of tuberculosis transmission to household contacts. Clin Infect Dis 2014; 58:765–74. 24368620 10.1093/cid/cit948 PMC3935504

[19] Corbett EL, Bandason T, Cheung YB Epidemiology of tuberculosis in a high HIV prevalence population provided with enhanced diagnosis of symptomatic disease. PLoS Med 2007; 4:e22. 17199408 10.1371/journal.pmed.0040022 PMC1761052

[20] Lawn SD, Wood R, De Cock KM, Kranzer K, Lewis JJ, Churchyard GJ Antiretrovirals and isoniazid preventive therapy in the prevention of HIV-associated tuberculosis in settings with limited health-care resources. Lancet Infect Dis 2010; 10:489–98. 20610331 10.1016/S1473-3099(10)70078-5

[21] Zachariah R, Bemelmans M, Akesson A Reduced tuberculosis case notification associated with scaling up antiretroviral treatment in rural Malawi. Int J Tuberc Lung Dis 2011; 15:933–7. 21682967 10.5588/ijtld.10.0666

[22] Dodd PJ, Looker C, Plumb ID Age- and sex-specific social contact patterns and incidence of Mycobacterium tuberculosis infection. Am J Epidemiol 2016; 183:156–66. 26646292 10.1093/aje/kwv160 PMC4706676

[23] Nobelprize.org. Robert Koch—Nobel Lecture. Stockholm, Sweden: Nobel Media, 2014 https://www.nobelprize.org/nobel_prizes/medicine/laureates/1905/koch-lecture.html. Accessed 12 August 2017.

[24] Shah NS, Yuen CM, Heo M, Tolman AW, Becerra MC Yield of contact investigations in households of patients with drug-resistant tuberculosis: systematic review and meta-analysis. Clin Infect Dis 2014; 58:381–91. 24065336 10.1093/cid/cit643 PMC3890332

[25] Zelner JL, Murray MB, Becerra MC Identifying hotspots of multidrug-resistant tuberculosis transmission using spatial and molecular genetic data. J Infect Dis 2016; 213:287–94. 26175455 10.1093/infdis/jiv387 PMC4690150

[26] Andrews JR, Morrow C, Walensky RP, Wood R Integrating social contact and environmental data in evaluating tuberculosis transmission in a South African township. J Infect Dis 2014; 210:597–603. 24610874 10.1093/infdis/jiu138 PMC4133578

[27] Yates TA, Khan PY, Knight GM The transmission of Mycobacterium tuberculosis in high burden settings. Lancet Infect Dis 2016; 16:227–38. 26867464 10.1016/S1473-3099(15)00499-5

[28] Andrews JR, Morrow C, Wood R Modeling the role of public transportation in sustaining tuberculosis transmission in South Africa. Am J Epidemiol 2013; 177:556–61. 23423215 10.1093/aje/kws331 PMC3657527

[29] Churchyard GJ, Fielding KL, Lewis JJ; Thibela TB Study Team A trial of mass isoniazid preventive therapy for tuberculosis control. N Engl J Med 2014; 370:301–10. 24450889 10.1056/NEJMoa1214289

[30] Telisinghe L, Fielding KL, Malden JL High tuberculosis prevalence in a South African prison: the need for routine tuberculosis screening. PLoS One 2014; 9:e87262. 24498059 10.1371/journal.pone.0087262 PMC3907552

[31] Johnstone-Robertson S, Lawn SD, Welte A, Bekker LG, Wood R Tuberculosis in a South African prison - a transmission modelling analysis. S Afr Med J 2011; 101:809–13. 22272961 PMC4538692

[32] Escombe AR, Huaroto L, Ticona E Tuberculosis transmission risk and infection control in a hospital emergency department in Lima, Peru. Int J Tuberc Lung Dis 2010; 14:1120–6. 20819256

[33] Escombe AR, Moore DA, Gilman RH Upper-room ultraviolet light and negative air ionization to prevent tuberculosis transmission. PLoS Med 2009; 6:e43. 19296717 10.1371/journal.pmed.1000043 PMC2656548

[34] Barrera E, Livchits V, Nardell E F-A-S-T: a refocused, intensified, administrative tuberculosis transmission control strategy. Int J Tuberc Lung Dis 2015; 19:381–4. 25859991 10.5588/ijtld.14.0680

[35] Cegielski JP, Griffith DE, McGaha PK Eliminating tuberculosis one neighborhood at a time. Am J Public Health 2013; 103:1292–300. 23078465 10.2105/AJPH.2012.300781 PMC3682594

[36] Fox GJ, Barry SE, Britton WJ, Marks GB Contact investigation for tuberculosis: a systematic review and meta-analysis. Eur Respir J 2013; 41:140–56. 22936710 10.1183/09031936.00070812 PMC3533588

[37] Shanaube K. The association between the magnitude of T-cell interferon-gamma responses to Mycobacterium tuberculosis specific antigens and risk of progression to tuberculosis in household contacts tested with QuantiFERON-TB Gold In-Tube assay [dissertation]. London, UK: London School of Hygiene and Tropical Medicine, 2014.

[38] Hanifa Y, Grant AD, Lewis J, Corbett EL, Fielding K, Churchyard G Prevalence of latent tuberculosis infection among gold miners in South Africa. Int J Tuberc Lung Dis 2009; 13:39–46. 19105877

[39] Cowie RL Short course chemoprophylaxis with rifampicin, isoniazid and pyrazinamide for tuberculosis evaluated in gold miners with chronic silicosis: a double-blind placebo controlled trial. Tuber Lung Dis 1996; 77:239–43. 8758107 10.1016/s0962-8479(96)90007-6

[40] Comstock GW, Ferebee SH, Hammes LM A controlled trial of community-wide isoniazid prophylaxis in Alaska. Am Rev Respir Dis 1967; 95:935–43. 6026165 10.1164/arrd.1967.95.6.935

[41] Nyboe J, Farah AR, Christensen OW. Report on chemotherapy pilot project (Tunisia 9). Geneva, Switzerland: World Health Organization, 1963.

[42] Ferebee SH Controlled chemoprophylaxis trials in tuberculosis. A general review. Bibl Tuberc 1970; 26:28–106. 4903501

[43] Horwitz O, Payne PG, Wilbek E Epidemiological basis of tuberculosis eradication. 4. The isoniazid trial in Greenland. Bull World Health Organ 1966; 35:509–26. 5335457 PMC2476023

[44] Horwitz O, Magnus K Epidemiologic evaluation of chemoprophylaxis against tuberculosis. Am J Epidemiol 1974; 99:333–42. 4596645 10.1093/oxfordjournals.aje.a121618

[45] Comstock GW, Baum C, Snider DE Jr Isoniazid prophylaxis among Alaskan Eskimos: a final report of the bethel isoniazid studies. Am Rev Respir Dis 1979; 119:827–30. 453704 10.1164/arrd.1979.119.5.827

[46] Rendleman NJ Mandated tuberculosis screening in a community of homeless people. Am J Prev Med 1999; 17:108–13. 10490052 10.1016/s0749-3797(99)00052-5

[47] Corbett EL, Bandason T, Duong T Comparison of two active case-finding strategies for community-based diagnosis of symptomatic smear-positive tuberculosis and control of infectious tuberculosis in Harare, Zimbabwe (DETECTB): a cluster-randomised trial. Lancet 2010; 376:1244–53. 20923715 10.1016/S0140-6736(10)61425-0 PMC2956882

[48] Ayles H, Muyoyeta M, Du Toit E; ZAMSTAR team Effect of household and community interventions on the burden of tuberculosis in southern Africa: the ZAMSTAR community-randomised trial. Lancet 2013; 382:1183–94. 23915882 10.1016/S0140-6736(13)61131-9

[49] Cavalcante SC, Durovni B, Barnes GL Community-randomized trial of enhanced DOTS for tuberculosis control in Rio de Janeiro, Brazil. Int J Tuberc Lung Dis 2010; 14:203–9. 20074412 PMC3812056

[50] Durovni B, Saraceni V, Moulton LH Effect of improved tuberculosis screening and isoniazid preventive therapy on incidence of tuberculosis and death in patients with HIV in clinics in Rio de Janeiro, Brazil: a stepped wedge, cluster-randomised trial. Lancet Infect Dis 2013; 13:852–8. 23954450 10.1016/S1473-3099(13)70187-7 PMC3899698

[51] Kranzer K, Afnan-Holmes H, Tomlin K The benefits to communities and individuals of screening for active tuberculosis disease: a systematic review. Int J Tuberc Lung Dis 2013; 17:432–46. 23485377 10.5588/ijtld.12.0743

[52] Small PM, Pai M Tuberculosis diagnosis–time for a game change. N Engl J Med 2010; 363:1070–1. 20825320 10.1056/NEJMe1008496

[53] Boehme CC, Nabeta P, Hillemann D Rapid molecular detection of tuberculosis and rifampin resistance. N Engl J Med 2010; 363:1005–15. 20825313 10.1056/NEJMoa0907847 PMC2947799

[54] Churchyard GJ, Stevens WS, Mametja LD Xpert MTB/RIF versus sputum microscopy as the initial diagnostic test for tuberculosis: a cluster-randomised trial embedded in South African roll-out of Xpert MTB/RIF. Lancet Glob Health 2015; 3:e450–7. 26187490 10.1016/S2214-109X(15)00100-X

[55] Theron G, Zijenah L, Chanda D; TB-NEAT team Feasibility, accuracy, and clinical effect of point-of-care Xpert MTB/RIF testing for tuberculosis in primary-care settings in Africa: a multicentre, randomised, controlled trial. Lancet 2014; 383:424–35. 24176144 10.1016/S0140-6736(13)62073-5

[56] Kendall EA, Shrestha S, Cohen T Priority-setting for novel drug regimens to treat tuberculosis: an epidemiologic model. PLoS Med 2017; 14:e1002202. 28045934 10.1371/journal.pmed.1002202 PMC5207633

[57] Zak DE, Penn-Nicholson A, Scriba TJ; ACS and GC6-74 cohort study groups A blood RNA signature for tuberculosis disease risk: a prospective cohort study. Lancet 2016; 387:2312–22. 27017310 10.1016/S0140-6736(15)01316-1 PMC5392204

[58] Rangaka MX, Cavalcante SC, Marais BJ Controlling the seedbeds of tuberculosis: diagnosis and treatment of tuberculosis infection. Lancet 2015; 386:2344–53. 26515679 10.1016/S0140-6736(15)00323-2 PMC4684745

[59] Guerra-Assuncao JA, Crampin AC, Houben RM Large-scale whole genome sequencing of M. tuberculosis provides insights into transmission in a high prevalence area. Elife. 2015; 4. 10.7554/eLife.05166 PMC4384740 25732036

[60] Knight GM, Griffiths UK, Sumner T Impact and cost-effectiveness of new tuberculosis vaccines in low- and middle-income countries. Proc Natl Acad Sci U S A 2014; 111:15520–5. 25288770 10.1073/pnas.1404386111 PMC4217399

